# Content analysis of press coverage during the H1N1 influenza pandemic in Germany 2009–2010

**DOI:** 10.1186/s12889-015-1742-1

**Published:** 2015-04-15

**Authors:** Sabine Husemann, Florian Fischer

**Affiliations:** Department of Public Health Medicine, School of Public Health, Bielefeld University, P.O. Box 100 131, 33501 Bielefeld, Germany

**Keywords:** H1N1, Influenza, Risk communication, Press coverage, Content analysis, Media

## Abstract

**Background:**

The H1N1 influenza pandemic occurred in Germany between April 2009 and August 2010. Pandemics often lead to uncertainty amongst the public and so risk communication on health-related issues is one of the key areas of action for health authorities and other healthcare institutions. The mass media may contribute to risk communication, so this study analysed press coverage during the H1N1 pandemic in Germany.

**Methods:**

A comprehensive analysis of the press coverage during the H1N1 pandemic was conducted in two steps. First, a temporal analysis was carried out of newspaper articles over the entire course of the pandemic, a total of 15,353 articles. The newspaper articles were obtained from the database Nexis. The total number of articles about the influenza pandemic during each individual week was plotted against the number of incident influenza cases during that week. Second, a quantitative content analysis of 140 newspaper articles from selected dates was conducted.

**Results:**

This study indicates that media awareness seems to be strongly related to the actual situation in the pandemic, because changes in the number of infected people were associated with nearly identical changes in the number of newspaper articles. Few articles contained information on the agent of the influenza or support measures. Information on vaccination was included in 32.9% of all articles. Almost half of the articles (48.6%) used case reports. Fear appeals were used in only 10.7% of the newspaper articles; 32.9% of the articles contained the message characteristic “self-efficacy”.

**Conclusions:**

The newspaper articles that were analysed in the content analysis included different information and message characteristics. The extent of information provided differed during the pandemic. As current research indicates, the use of message characteristics such as fear appeals and self-efficacy, which were also included in the analysed newspaper articles, can help to make health messages effective.

## Background

The H1N1 influenza pandemic, also known as swine flu, was the first pandemic of the 21st century. The pandemic was a major challenge for public health due to its long duration and the need to establish effective countermeasures such as improved personal hygiene and vaccination [1]. The first three cases of H1N1 influenza occurred in Germany in April 2009 [2]. At the end of September that year, the first death attributable to the consequences of an infection with the H1N1 virus occurred [3]. In the 47th week of 2009, the pandemic wave reached its highest level, with 45,000 cases reported during the week. Almost all the deaths associated with this pandemic occurred during this period. About 1.8 to 3.5 million additional medical consultations were estimated to have occurred in the 2009–2010 influenza season [1]. No new H1N1 cases attributable to this pandemic were reported after August 2010. The age distribution of influenza cases attributable to the H1N1 pandemic is similar to that of seasonal influenza. However, deaths attributable to the pandemic were mainly in infants (0.44 per 100,000 infants [95% CI: 0.16–0.95]) and adults aged between 35 and 59 years (0.42 per 100,000 inhabitants [95% CI: 0.35–0.50]). The cumulative mortality was 0.22 per 100,000 inhabitants (95% CI: 0.16–0.30) in the group aged 15–34 years, and 0.24 per 100,000 inhabitants (95% CI: 0.18–0.32) in those aged 60 and over. By contrast, about 90% of seasonal influenza-related deaths occur in those aged 60 and over. The overall mortality was 0.31 (95% CI: 0.27–0.35) per 100,000 inhabitants [4].

By 20 April 2010, a total of 226,137 people had been infected with the H1N1 virus, 7,882 cases had been admitted to hospital because of it and 253 H1N1-associated deaths were confirmed in Germany. The actual number of H1N1 patients is believed to be much higher because of unreported cases, because not all people with influenza sought care from the healthcare system, and only formally-diagnosed cases were reported to the Robert Koch Institute (RKI) [1,5,6].

Crisis management during the pandemic concentrated primarily on promoting the use of vaccination and hygiene measures, and adaptation of the surveillance system. For example, notification of potential or actual cases of the disease to the health authorities became mandatory for physicians from May 2009. After the number of patients increased worldwide, the World Health Organization (WHO) declared a level six pandemic, the highest possible, on 11 June 2009. This was followed by preliminary considerations about the preparation of vaccines and recommendations for vaccination. In July 2009, the federal states of Germany agreed to order 50 million doses of vaccine against H1N1 influenza. According to the health authorities, 100 million doses of the vaccine should have been made available by October 2009. School children, pregnant women, the chronically ill and hospital workers were prioritised for vaccination [5].

### Influence of mass media on health behaviour

Uncertainty amongst the population increases with growing complexity of health risks. Risk communication about health-related issues is therefore one of the key areas of action for health authorities and other healthcare institutions [7,8]. According to the WHO, risk communication is a “process which aims to help stakeholders define risks, identify hazards, assess vulnerabilities and promote community resilience” [9]. The dissemination of information to the public about health risks and events is, therefore, an essential component. The mass media may contribute to this, essentially acting as a transmitter of information. Risk communication about health-related topics has two aims. First, politicians, the public and the scientific community should receive activity-oriented information about adequate measures to prevent the acquisition of infectious diseases. Second, appropriate information strategies should be developed to educate the public about health risks, and support the establishment of effective countermeasures [10]. Risks are closely linked to human activities and the controllability of future events. This controllability is not linked to the perceived level of threat: a risk that is perceived as serious may also be perceived as more or less controllable, depending on perceptions about protective measures [11].

Risk perception and the response to a risk are dependent on several factors, such as personal experience, values and cultural background [8]. The complexity of subjective perceptions of risk and the difficulty of influencing these perceptions are major barriers in risk communication. Particularly from a health psychological point of view, perceptions of risk and personal vulnerability are highly relevant and have to be considered in communicating information about preventive measures [10,12].

This study made a content analysis of newspaper articles published during the H1N1 pandemic of 2009–2010 in Germany. The aim was to show how press coverage changed over the course of the pandemic. The number of disease cases was plotted against the number of newspaper articles in every week during the pandemic. The content and message characteristics in the context of risk communication in the press coverage were systematically assessed. The objective was to identify how far information provided by the media was linked to the current situation. It also identified what types of information were mainly published by the media. These results are needed to make recommendations for effective risk communication of health-related topics.

## Methods

This comprehensive analysis of the press coverage during the H1N1 pandemic involved two steps. First, a temporal analysis was conducted of the 15,353 newspaper articles published over the course of the pandemic and, second, a quantitative content analysis of 140 newspaper articles from selected dates was undertaken. The newspaper articles were obtained from the database Nexis (www.nexis.com), which provides access to national and international press reports and newspaper articles. For the analysis, all newspaper articles listed in Nexis from regional and national newspapers in Germany, both daily and Sunday papers, were used. The search algorithm “H1N1 OR swine flu OR new influenza” was applied, since this variant of influenza was commonly called ‘new influenza’ in Germany. An automatic duplicate analysis was performed.

All articles published during the observation period of 27 April 2009 to 22 August 2010 were included. The observation period was one week longer than the pandemic phase. This interval was chosen to determine changes in the design and content of newspaper articles after the end of the pandemic phase. The total number of newspaper articles about the influenza pandemic published each week was plotted against the number of influenza cases for the week. Information on disease cases came from the RKI’s “SurvStat” database.

A content analysis of selected newspaper articles was then performed for a limited time period during the pandemic. The observation period of 4 June to 16 July 2009 was chosen, because on 11 June, the WHO declared H1N1 influenza to be a global pandemic (phase 6), which drew a lot of attention to the issue [13]. A total of seven dates were evaluated. The selected dates were set as Thursdays (4 June, 11 June, 18 June, 25 June, 2 July, 9 July and 16 July 2009). Three further dates were selected from the three weeks at the end of the pandemic (5 August, 12 August and 19 August 2010). Because there were very few articles at the end of the pandemic (n = 16), coverage for these three dates was summarised and used mainly to illustrate changes over time in the discussions about communication and reporting by public institutions.

Through automatic duplicate analysis, 50 of the initial 241 articles identified were excluded. A further 51 articles were excluded because they met one or more of the following exclusion criteria:

- occurrence of keywords, but a different focus of the article;
- only a short note on the front page; or
- a readers’ poll.

**Figure 1:**
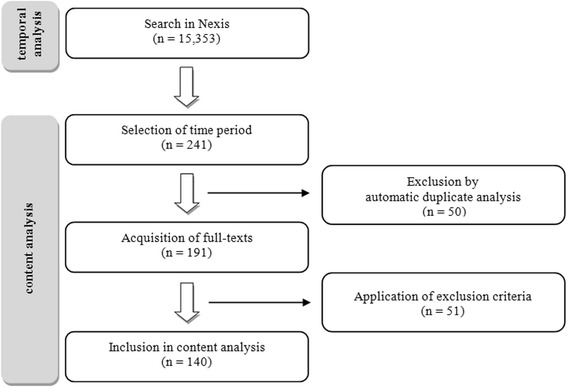
Flow diagram for the systematic analysis of newspaper articles.

Overall, 140 articles were considered in the content analysis (Figure 1). A categorical framework was created to capture aspects of both content and subjectively-assessed message characteristics. The message characteristics were based on theories and models to explain the effects on health behaviour of health communication via the mass media [14]. These include *fear appeal* approaches, characterised by descriptions of the negative consequences of health risks or health behaviour. Fear appeals are often used in health messages to initiate changes in attitudes and behaviour in recipients. Although this strategy is still controversial, fear appeals are often used to confront a targeted population with the relevant risk [15].

Several message characteristics influence risk perception within a population. The message characteristic “*threat*” is based on the assumption that threatening messages are particularly well-suited to changing attitudes and behaviours. In a variety of models and approaches to health behaviour, threat is understood as a dimension of the negative consequences of a health risk and concern about its consequences. It is important to distinguish between the objective and perceived threat, because perceived risks may differ from objective threat probabilities [16].

Highlighting measures that people can take to protect themselves against certain health risks is considered to be an important aspect of strengthening the sense of “*self-efficacy*” in the population [17]. People need to have a sense of self-determination and controllability of their environment to respond appropriately to risks at a cognitive, emotional and behavioural level. Self-efficacy is described as confidence in one’s own personal strengths and skills, which motivates an individual to initiate certain actions. The construct of self-efficacy is incorporated into many known models and theories of health behaviour, and represents a significant factor in the explanation of behaviour and attitude changes [18].

Another message characteristic is the “*evidence description*”, which is an important stylistic tool in communicating health risks. The description of the evidence is subdivided into two components: 1) statistical or summary descriptions and 2) episodic, anecdotal or narrative descriptions of reality. A summary of evidence is characterised by the use of statistical information. Episodic evidence includes the use of case studies or other personal testimonials. Both components are intended to serve as proof of the statements of the communicator. Statistical and episodic evidence differ in several aspects, such as specificity, emotionality or representativeness [17,19,20].

A descriptive analysis of newspaper articles using the statistical program SPSS version 19 was performed. The data were analysed using descriptive frequency analyses and cross tables.

## Results

### Temporal analysis of press coverage

**Figure 2:**
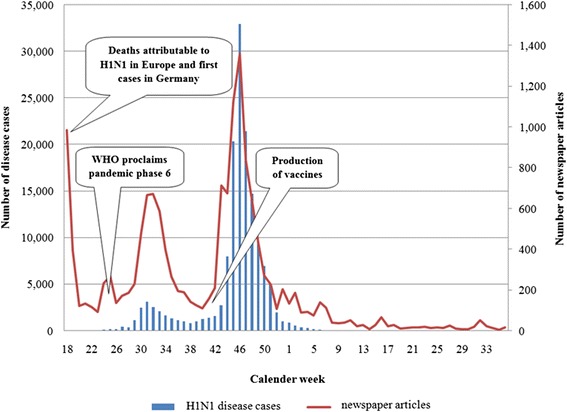
Number of newspaper articles and number of cases of disease throughout the course of the pandemic in 2009 and 2010.

Figure 2 shows the time course of the number of influenza cases and press coverage throughout the pandemic, and highlights some events that may have increased the likelihood of media awareness. Changes in the number of influenza cases were associated with nearly identical changes in the number of newspaper articles. This suggests that media awareness and press coverage was strongly related to the actual situation. During the 24th calendar week, the first influenza cases were reported in Germany. However, newspaper articles about influenza had appeared on 27 April 2009 (calendar week 18), before the first cases were reported in Germany. During the 22nd calendar week, the number of newspaper articles initially increased. It was during this period that pandemic phase 6 was declared by the WHO. From calendar week 40 to 46, more newspaper articles about influenza appeared, the number of disease cases increased, and plans for vaccination began. Calendar week 46 saw a peak in the number of both cases and newspaper articles. After this week, the number of both steadily declined to reach a comparatively low level by 2010 calendar week 9, where it remained.

### Content analysis of newspaper articles

Only newspaper articles from the seven selected dates during the main phase and the three dates during the final stage of the pandemic were used in the content analysis. Articles published on the three dates of the final stage of the pandemic were summarised as there were so few. A total of 140 newspaper articles were categorised in the content analysis (see Table 1).

**Table 1:** **Categorisation of newspaper articles**

**Categories**	***4 June 2009***	***11 June 2009***	***18 June 2009***	***25 June 2009***	***2 July 2009***	***9 July 2009***	***16 July 2009***	***5, 12 and 19 August 2010***	***∑***
**Number of articles**	**13**	**18**	**21**	**6**	**20**	**10**	**36**	**16**	**140**
	**% (n)**	**% (n)**	**% (n)**	**% (n)**	**% (n)**	**% (n)**	**% (n)**	**% (n)**	**% (n)**
**Information on disease/virus**									
H1N1	7.7 (1)	27.8 (5)	14.3 (3)	33.3 (2)	15.0 (3)	40.0 (4)	16.7 (6)	0 (0)	17.1 (24)
Influenza and H1N1	0 (0)	5.6 (1)	4.8 (1)	0 (0)	0 (0)	0 (0)	8.3 (3)	0 (0)	3.6 (5)
No information	92.3 (12)	66.7 (12)	80.1 (17)	66.7 (4)	85.0 (17)	60.0 (6)	75.0 (27)	100 (16)	79.3 (111)
**Facts and figures**									
Cases	0 (0)	0 (0)	4.8 (1)	0 (0)	0 (0)	0 (0)	0 (0)	0 (0)	0.7 (1)
Deaths	23.1 (3)	44.4 (8)	38.1 (8)	83.3 (5)	60.0 (12)	40.0 (4)	22.2 (8)	6.3 (1)	35.0 (49)
Cases and deaths	23.1 (3)	27.8 (5)	4.8 (1)	16.7 (1)	10.0 (2)	20.0 (2)	22.2 (8)	12.5 (2)	17.1 (24)
Not applicable	53.8 (7)	27.8 (5)	52.4 (11)	0 (0)	30.0 (6)	40.0 (4)	55.6 (20)	81.3 (13)	47.1 (66)
**Information on…**									
Course of the pandemic	15.4 (2)	66.7 (12)	47.6 (10)	33.3 (2)	50.0 (10)	60.0 (6)	52.8 (19)	68.8 (11)	51.4 (72)
Severity of disease	15.4 (2)	27.8 (5)	28.6 (6)	66.7 (4)	30.0 (6)	60.0 (6)	38.9 (14)	6.3 (1)	31.4 (44)
Support measures	7.7 (1)	16.7 (3)	23.8 (5)	16.7 (1)	25.0 (5)	20.0 (2)	13.9 (5)	0 (0)	15.7 (22)
Protective measures	7.7 (1)	11.1 (2)	23.8 (5)	33.3 (2)	10.0 (2)	50.0 (5)	33.3 (12)	12.5 (2)	22.1 (31)
Vaccination	0 (0)	22.2 (4)	23.8 (5)	16.7 (1)	10.0 (1)	52.8 (19)	87.5 (14)	100 (1)	32.9 (49)
Economic consequences	0 (0)	0 (0)	19.0 (4)	0 (0)	15.0 (3)	10.0 (1)	11.1 (4)	25.0 (4)	11.4 (16)
Political decisions	15.4 (2)	72.2 (13)	33.3 (7)	50.0 (3)	35.0 (7)	60.0 (6)	63.9 (23)	68.8 (11)	51.4 (72)
**Criticism regarding political decisions**	0 (0)	0 (0)	9.5 (2)	16.7 (1)	5.0 (1)	20.0 (2)	8.3 (3)	68.8 (11)	14.3 (20)
**Origin of information**									
Institution	46.2 (6)	16. (3)	14.3 (3)	16.7 (1)	45.0 (9)	30.0 (3)	22.2 (8)	6.3 (1)	24.3 (34)
Expert	0 (0)	5.6 (1)	19.0 (4)	50.0 (3)	10.0 (2)	20.0 (2)	27.8 (10)	18.8 (3)	17.9 (25)
Institution and expert	30.8 (4)	44.4 (8)	38.1 (8)	16.7 (1)	25.0 (5)	40.0 (4)	36.1 (13)	18.8 (3)	32.9 (46)
Other	7.7 (1)	0 (0)	0 (0)	0 (0)	0 (0)	0 (0)	0 (0)	12.5 (2)	2.1 (3)
Not applicable	15.4 (2)	33.3 (6)	28.5 (6)	16.7 (1)	20.0 (4)	10.0 (1)	13.9 (5)	43.8 (7)	22.9 (32)
**Case reports**									
Yes, children	0 (1)	5.6 (1)	4.8 (1)	0 (0)	10.0 (2)	0 (0)	2.8 (1)	0 (0)	3.6 (5)
Yes, adults	46.2 (6)	0 (0)	14.3 (3)	0 (0)	25.0 (5)	10.0 (1)	5.6 (2)	0 (0)	12.1 (17)
Yes, small group	15.4 (2)	55.6 (10)	33.3 (7)	100 (6)	35.0 (7)	40.0 (4)	27.8 (10)	0 (0)	32.9 (46)
No case reports	38.5 (5)	38.9 (7)	47.6 (10)	0 (0)	30.0 (6)	50.0 (5)	63.9 (23)	100 (16)	51.4 (72)
**Message characteristics**									
Fear appeal	0 (0)	44.4 (8)	0 (0)	0 (0)	10.0 (2)	20.0 (2)	8.3 (3)	0 (0)	10.7 (15)
Threat	15.4 (2)	83.3 (17)	23.8 (5)	16.7 (1)	25.0 (5)	30.0 (3)	33.3 (12)	0 (0)	30.7 (43)
Self-efficacy	23.1 (3)	16.7 (3)	48.5 (10)	33.3 (2)	30.0 (6)	30.0 (3)	50.0 (18)	6.3 (1)	32.9 (46)
**Evidence description**									
Case report	53.8 (7)	0 (0)	38.1 (8)	16.7 (1)	40.0 (8)	20.0 (2)	22.2 (8)	0 (0)	24.3 (34)
Statistics, facts and figures	23.1 (3)	11.1 (2)	23.8 (5)	0 (0)	15.0 (3)	10.0 (1)	19.4 (7)	12.5 (2)	16.4 (23)
Case report and statistics	7.7 (1)	61.1 (11)	9.5 (2)	83.3 (5)	30.0 (6)	20.0 (2)	11.1 (4)	0 (0)	22.1 (31)
Not applicable	15.4 (2)	27.8 (5)	28.6 (6)	0 (0)	15.0 (3)	50.0 (5)	47.2 (17)	87.5 (14)	37.1 (52)

The percentage of articles is arranged in columns for each category. The absolute number of articles is mentioned in brackets.

Only 17.1% of all articles considered in the quantitative content analysis (n = 24) contained information on the influenza virus in general or H1N1 in particular, and only 3.6% of articles (n = 5) contained information about both. In most articles (79.3%; n = 111), there was no information at all about H1N1 influenza, or influenza in general. Overall, half of the articles included data on the H1N1 pandemic (52.8%; n = 74). The extent of data on the H1N1 pandemic did not differ between the selected dates. 31.4% (n = 44) of the articles contained information about the severity of the disease attributable to the H1N1 influenza virus. The number of articles containing such information increased slightly over time.

Information on support measures was found in only 15.7% of the articles (n = 22). This information included telephone helplines, information meetings and brochures. The number of articles containing such information remained relatively constant within the main phase and reached a maximum of 23.8% (n = 5) of articles on the third selected date (18 June 2009). In 22.1% of newspaper articles (n = 31), protective measures were outlined.

Information on vaccination was included in 32.9% of all articles (n = 46). This included vaccination recommendations, information on the risks and benefits of vaccination against the H1N1 virus, and the cost of vaccines. Across the seven dates in the main phase, the prevalence of information on vaccination increased from no articles at the beginning of the selected time period to 19 (52.8%) on the last date (16 July 2009).

Details about the origins of the information in the newspaper articles were found in almost all of them. Only 22.9% (n = 32) included no evidence to allow conclusions to be drawn on the source of the information. In 24.3% (n = 34), the information came from institutions such as the RKI or the WHO. Expert opinions were also prominent sources of information (17.9%; n = 25), and 32.9% (n = 46) of the articles contained information from both institutions and experts.

Almost half of the articles (48.6%, n = 68) included case reports. These were most often descriptions of the impact of H1N1 influenza on small groups (32.9%; n = 46), for example, schools or families affected or threatened by infection. Individual cases were described in only 3.6% of the articles (n = 5), and 17 articles (12.1%) were about adults.

Information on political decisions was included in more than half of the articles (51.4%; n = 72), and this increased over time. On the first date analysed during the main phase (4 June 2009), information on political decisions was included in only 15.4% of the articles (n = 2). By the last date of the main phase (6 July 2009), 63.9% (n = 23) of the articles reported on these issues. A similar trend was seen in the description of economic consequences. At the beginning of the observation period, there were no articles about this, but on the last date analysed during the main phase, there were four articles (11.1%) that covered this issue.

Criticism regarding political decisions about proposed or actual measures, and of communication on the pandemic by governing bodies, appeared in only nine newspaper articles (7.3%) during the main phase. In contrast, during the final phase, criticisms were raised in 11 articles (68.8%).

Classification into categories of message characteristics was made from a subjective impression of the newspaper articles. Fear appeals were found in only 10.7% of articles (n = 15). ‘Threat’ was present in 30.7% (n = 43) of the articles. This was particularly marked on the second selected date (11 June 2009), when the content was perceived as threatening in 83.3% (n = 17) of the articles. This may be related to the fact that pandemic phase 6 was declared by the WHO on that date. Apart from that, the occurrence of this message characteristic remained relatively constant, at between 15% and 35% of articles.

Around one third, 32.9% (n = 46) of the newspaper articles contained the message characteristic “self-efficacy”. The proportion of articles strengthening the feeling of self-efficacy tended to increase over the seven dates in the main phase. On the last date (16 July 2009), 50.0% (n = 18) of the articles included this message characteristic, while on the first date, it was only 23.2% (n = 3).

The message characteristic “evidence description” can be divided into the following four characteristics: 1) case report, 2) statistics, facts and figures, 3) case report and statistics, and 4) not applicable. Case reports were included in 34 out of 140 articles (24.3%). Statistical information, such as the number of cases or deaths attributable to the H1N1 influenza, were included in 23 articles (16.4%), and 31 articles (22.1%) included both statistical information and case reports. Evidence descriptions of some kind were used in more than half of the articles (62.8%; n = 88).

## Discussion

The H1N1 pandemic of 2009–2010 was highly visible in the media [21]. A lot of newspapers covered this topic several times during the period, many in prominent positions such as the title page. The results of this study indicate that the extent of press coverage on aspects of the H1N1 pandemic was closely linked to single events, as shown in Figure 2. This may be connected to the assumptions of the agenda-setting approach, which states that the media can influence what is discussed by the public and the extent to which it is discussed. The initially high number of newspaper articles before the beginning of the observation period for the content analysis is probably linked to the fact that infections and deaths attributable to the H1N1 virus had already occurred in other European countries. The first cases of disease caused by an infection with the H1N1 virus occurred in Germany at this time. The reason for the decrease in the number of newspaper articles after this initial period is probably because cases were comparatively rare in Germany at the beginning. The declaration of pandemic phase 6 on 11 June 2009 led to an increase in the number of newspaper articles. After the coverage decreased again, the decision to start mass production of vaccines and the planning of nationwide vaccination campaigns caused a renewed increase in the number of newspaper articles. At this time, the number of infections in Germany and the number of newspaper articles dealing with the H1N1 pandemic both increased significantly.

If the media coverage includes many different articles on a topic, this may lead to the dissemination of incomplete, incorrect and contradictory information, which can increase uncertainty amongst the public. The growing volume of criticisms by the end of the pandemic suggests that, even during the H1N1 pandemic, conflicting information had led to uncertainty [22]. Effective dissemination of health messages requires close knowledge of the recipients. Their level of awareness needs to be taken into account in developing messages. In particular, health messages during crisis situations should provide consistent information, requiring collaboration between healthcare leaders. Consensus in reporting can help to gain the trust of the population [23].

Particularly at the beginning of a pandemic, descriptions of protective measures are essential, because at this time there is not usually enough vaccine available. The content analysis of newspaper articles showed that overall, only 31% included such information. This is also consistent with the critical assessments in the literature [24]. There was probably insufficient knowledge about hygiene in the general population, because hygiene measures were not discussed very often in the media [24].

In a survey of vaccination use against the H1N1 influenza, a significant decline in vaccination in Germany in November 2010 was observed [25,26]. In a telephone-based cross-sectional survey by the RKI, willingness to receive a vaccination was compared with the epidemiological data on cases attributable to the H1N1 virus. The results of this survey showed that the decline in H1N1 cases also led to a decline in the perception of risk. In early February 2010, around 70% of respondents reported that they felt sufficiently informed about the H1N1 influenza virus. Respondents obtained their knowledge about the newly-emerged virus mainly through television, radio and newspapers. This emphasises the relevance of these media to risk perception. Just over half of the respondents (55%) felt insecure about vaccination against the H1N1 virus because of reports in the media. By January 2010, there was very little willingness to receive the vaccination. Despite existing vaccination recommendations, about 80% of the respondents judged that vaccination was no longer necessary. Even the groups designated as being at risk only had a vaccination rate of about 15% [25,26].

The decline in demand for vaccinations has been attributed to the communication process. For example, inconsistent communication about the responsibilities of the federal and state governments could have led to the decrease. The contradictory opinions of experts about the vaccination may have caused uncertainty, and therefore led to its rejection [24].

Message characteristics (fear appeals, threat, self-efficacy, evidence descriptions) can help to make health messages more effective. A sense of self-efficacy, credibility and sustainability are crucial for behavioural changes related to vaccinations [27]. It should be noted, however, that the correct use of messages is more important than their volume. For example, fear appeals or threats are best combined with self-efficacy messages to achieve a positive impact on health behaviour and attitudes. The excessive use of all these stylistic devices can lead to avoidance behaviour and displacement and thus generate negative effects. The reporting of health risks should therefore aim to convince recipients that a simple change in behaviour can significantly minimise the risks [15].

The message characteristics “self-efficacy” (32.9%; n = 46) and fear appeals (10.7%; n = 15) were rarely used in newspaper articles during the H1N1 pandemic. Protection or support measures were discussed in articles (22.1%; n = 31), however, which could increase the sense of self-efficacy. Promotion of self-efficacy is a central aspect of health communication [28].

The locus of control is also an important aspect. The existing empirical research suggests that a distinct locus of control will support health-promoting behaviour. Our findings indicate that self-efficacy messages were used more frequently than threats. This is important for changes in health behaviour, because messages that are highly threat-oriented, without directly observable self-efficacy components, can cause defensive and avoidance strategies [17].

The component of evidence description permits several conclusions. The press coverage during the H1N1 pandemic was characterised both by the description of individual cases and through the use of data and facts. Communication via mass media often includes appeals to the emotions of users. The use of case reports should be regarded as an effective strategy to illustrate health issues and to provide better health information. Statistical data were less frequently used than case reports. It seems likely that journalists assumed that a proportion of the population would be overwhelmed by having to process statistical information [11].

The use of an incomprehensible form to communicate information could be a barrier to the perception of health messages. Too many facts and figures is regarded as counterproductive to effective health communication in crisis situations and may even lead to avoidance behaviour [28,29]. Since risk communication depends on statistical data, previous studies have recommended using frequencies instead of percentages, because they are usually easier to understand [30].

The fact that not as many people were affected as was expected during the pandemic may also have led to criticisms of various measures that were undertaken and, therefore, misjudged by the public. The RKI emphasised, however, that all of these steps were necessary, because the potential risk from the unexpectedly severe disease course in people less than 60 years old was hard to assess. It was also reasonable, therefore, to reduce or stop several measures linked to infection control and surveillance at a later point in time, to avoid any unexpected spread of the influenza. In comparison with other European countries, Germany had fewer deaths attributable to infection with the H1N1 virus. Because of the different capacities of the federal states in Germany and the respective spread of the H1N1 virus, there were in some cases large regional differences between the measures taken, which may have contributed to uncertainty and confusion amongst the public. Krause et al. [1] referred to the partially-incomplete coordination and cooperation between counties and federal authorities. The fact that some federal states introduced more complex measures than others may have led to the impression amongst the public that these procedures were uncoordinated and disorganised. Similarly, the failure to meet the information needs of the population may have led to an underestimation of the situation amongst the public [1].

### Limitations

The results of this quantitative content analysis are subject to some limitations. First, only articles from newspapers listed in Nexis could be included. It is likely that other articles on this topic were published, but could not be identified using the search algorithm. The content analysis covered only one day per week, which was used to represent the coverage throughout the week. It is therefore only possible to detect a general trend and no changes within weeks. The categorisation, particularly in terms of the message characteristics, is partially based on subjective judgements.

## Conclusions

The newspaper articles that were analysed in the content analysis included different information and message characteristics. The extent of information provided differed over time during the pandemic. Previous research suggests that the use of message characteristics such as fear appeals and self-efficacy, which were included in the analysed newspaper articles, can help to make health messages more effective. This analysis of the press coverage during the H1N1 pandemic points to several possible recommendations for better risk communication in health-related issues. For vaccine-preventable infectious diseases that spread quickly, the benefits as well as risks of a vaccination have to be communicated transparently. Even basic knowledge about hygiene measures has to be highlighted more clearly.

## Abbreviations

RKI: Robert Koch Institute
WHO: World Health Organization
